# Enhancing Care for Multimorbidity in Adults with Neurodevelopmental Disorders

**DOI:** 10.1007/s40737-023-00334-7

**Published:** 2023-02-21

**Authors:** Krishna Prasad Muliyala, R. Sujai, Jagadisha Thirthalli

**Affiliations:** Psychiatric Rehabilitation Services (PRS), Department of Psychiatry, National Institute of Mental Health & Neurosciences (NIMHANS), Bengaluru, 560029 India

## Introduction

Neurodevelopmental disorders (NDD) are a group of disorders that affect brain development, clinically with an onset usually in childhood and cause a wide range of disabilities. Although a broad range of disabilities are covered under the umbrella term of NDD, for this article we restrict ourselves to autism and disorders of intellectual development (DID) in adults, the most disabling of the NDDs among adults. While the services and interventions for children with NDD has grown over the years and are structured in many countries, the same has not translated for transitioning adults with NDD and across the lifespan. In most countries, adults with NDD are provided care mostly in routine adult psychiatric services that are not suited for managing their complex needs. Both the conditions follow a steady course continuing into adulthood, although there could be maturational changes. It is important to consider, however, that distinctions between conditions may not be easy and there may be overlap. 

The term multimorbidity has been used when two or more chronic health conditions co-exist in an individual (Skou et al. 2022). Multimorbidity has been associated with poor quality of life, disability, and premature mortality. There are several challenges in detecting physical and mental health morbidities in adults with NDD. While in the general population multimorbidity prevalence increases with increasing age, in people with NDD, the prevalence is higher and occurs at much earlier age (Hossain et al. 2020). Persons with both autism and DID are likely to be affected to a greater extent. 

### Challenges in Detecting Morbidity in Adults with NDD

Persons with NDD maybe overwhelmed and uncomfortable in crowded general hospital settings and the source of distress could be undetected sensory issues. Adults with neurodevelopmental disorders may not co-operate for formal medical or mental status examination. In persons with autism, where social communication problems are the core feature, there are challenges in detection of health conditions. In persons with NDD, poor ability to mentalize or introspect leads to challenges in reporting psychological experiences like anxiety, sadness, delusions or hallucinations. Physicians may not be able to communicate effectively because of lack of adequate training in effective communication with persons with NDD. Deficiency of human resources and high volumes in routine adult psychiatry/general medical services result in under-detection of other health conditions. Identification of some conditions may be difficult because of confounding—for example, autism and psychosis, causing diagnostic dilemmas. If developmental history is not reliably available for adults with NDD, the core features of autism can be mistaken for psychosis. It is important to recognise changes from the baseline when the information is available. Features such as formal thought disorder have been noted to be prevalent in adults with autism (Cochran et al. 2013).

### Psychiatric Conditions in Adults with NDD

Several psychiatric conditions have been reported to have higher prevalence in persons with autism when compared to neurotypical persons. The wide range of prevalence reported in the literature makes it hard to estimate their exact prevalence. For example, prevalence of psychotic disorders has been noted to vary from 4 to 67% (Hossain et al. 2020). A recent meta-analysis has reported the rate of schizophrenia and related disorders in autism to be 9.4% (Varcin et al. 2022). Mood disorders (including bipolar disorder) have been noted in as high as nearly every third person with autism (Hossain et al. 2020). The rates of ADHD and common mental disorders are similarly higher among persons with NDD than among the general population (Lai et al. 2019). A higher rate of psychiatric comorbidity has been observed in DID compared to the general population (Mazza et al. 2020). The rates have been noted to be lower in profound DID compared to mild, moderate or severe DID (Mazza et al. 2020).The burden of psychiatric morbidity is even higher in persons who have both DID and autism (Peña-Salazar et al. 2022). Persons with DID who have addictive disorders have at least one psychiatric comorbidity in more than 75% of them (Lin et al. 2016).

The methods that are used to diagnose psychiatric conditions in typically developed adults may not be suitable for adults with NDD. Problem behaviours in persons with NDD may have several origins and need not necessarily be indicative of mental illness. Impaired verbal abilities preclude clarifications about delusions or hallucinations. Idiosyncrasies in persons with autism can be confused with delusions. However, behavioural equivalents can correspond to symptoms of mental illness and these can enhance the accuracy of diagnosis. In general, new onset disorganization, negative symptoms or hallucinatory behaviour can be interpreted as symptoms of psychosis in NDD (Cochran et al. 2013). Persistent psychomotor agitation, aggression, and decreased sleep are predictive of mania in adults with severe/profound DID (Matson et al. 2007). Self-injurious behaviours, aggression, disruptive behaviours, screaming episodes and tantrums have been associated with depression in severe to profound DID (Eaton et al. 2021). Caution needs to be exercised, as evidence for challenging behaviours as behavioural equivalents of psychosis or mood disorders has been mixed/contradictory (Tsiouris et al. 2003). Serial behavioural observation, longitudinal follow up, and enhancing non-verbal communication may aid in accurately diagnosing. Structured assessment instruments have been developed for persons with DID, and, along with the burgeoning number of tools, criteria have evolved for evaluating the instruments as well (Zeilinger et al. 2013).

### Medical Conditions in Adults with NDD

Medical disorders such as epilepsy, sleep disorders, gastrointestinal disorders, allergy/immunological conditions, metabolic disorders, musculoskeletal disorders and hormone dysfunction are more common in adults with autism, compared to the general population (Davignon et al. 2018). While they deserve adequate quality of care, the barriers related to medical history and examination impose limitations. Medical conditions could also be construed to be a part of the clinical phenotype in many cases. Similarly high rates of medical comorbidities have been observed in persons with DID. For example, persons with Down syndrome are at increased risk of hypothyroidism, metabolic disorders, ear infections and Alzheimer’s dementia. Overall, in DID, epilepsy, ear and eye disorders, cerebral palsy, obesity, osteoporosis, congenital heart defects, and thyroid disorders have the highest prevalence amongst physical health conditions (Anderson et al. 2013). Besides being symptomatic of the clinical phenotype, several factors contribute to health disparities in care, including lack of access to quality medical care, healthcare system that is unprepared to provide care to persons with NDD and reasons such as poverty, race, and gender.

### Polypharmacy in Adults with NDD

Polypharmacy and inappropriate medication use are high in adults with NDD. More than one third of adults with DID are exposed to polypharmacy and nearly a quarter are exposed to antipsychotic polypharmacy, increasing the risk adverse events and drug–drug interactions (McCarthy and Chaplin 2022). In fact, the presence of DID has been reported to be a significant predictor of polypharmacy. Many manifestations of the core features of autism, mistaken for other psychopathology, may not respond to medications. To address this issue, National Health Service in England initiated the project, “stopping over medication of people with a learning disability, autism or both with psychotropic medicines (STOMP)” (NHS England, 2023). STOMP also envisages the use of psychological-focused interventions, sensory-focused adaptations and other such alternatives as a first line for problem behaviours. To our knowledge there are no such policies in the low- and middle-income countries (LAMIC). Effective implementation and audit of STOMP should provide a greater knowledge base to develop these in low resource settings, where trained human resource to deliver such interventions are sparse.

### “Missed” Adults with NDD

In certain situations, NDD may be missed until adulthood. This often occurs at late adolescence or early adulthood, when the social demands exceed the abilities of the person. Not infrequently, there is lack of informants to provide accurate developmental history, and, the information provided by the individual may be inadequate. In the case of persons with high functioning autism, the symptoms or social difficulties may be masked or camouflaged because of the higher IQ or cognitive abilities. The problem of delayed identification of NDD is more likely among women. Due to the overlap of symptoms and psychopathology in this context, misdiagnosis of personality disorders, OCD or psychosis may be ascribed by clinicians (Fusar-Poli et al. 2022). Revision of diagnostic criteria (ICD 11 and DSM 5) may add a group of patients who are from a missed generation because the previous diagnostic criteria had not picked them up. This may increase with greater awareness in the general population.

Autism may also be misdiagnosed as DID. An exactly opposite scenario is the challenge in diagnosing autism in persons with intellectual disability or other sensory disabilities. This may be true with greater level of intellectual disability or with specific disabilities such as visual, hearing impairment and multiple disabilities. Observation of autism in deaf blindness (O-ADB) and its modification observation of autism in people with sensory and intellectual disabilities (OASID) offer structured means of clinical distinction with satisfactory psychometric properties (de Vaan et al. 2016). Tools such as these that use observation and do not rely only on a questionnaire are likely to aid valid diagnosis.

### Importance of a Lifespan Approach

Life expectancy has been increasing in the general population as also for persons with DID. Accelerated aging has been observed in Down syndrome (Alldred et al. 2021) and premature mortality has been observed in both DID (Hirvikoski et al. 2021) and autism (Hirvikoski et al. 2016) when compared to the general population. While health services for aging adults in the general population has grown over the years even in the LAMIC and there is a recent progress in the literature base for aging in DID, the understanding of mid or late life changes in persons with autism has lagged. In a study that looked at persons aged 30–64 years over a period of 5 years, there was an increased risk of early onset dementia in adults with autism (4%), while the risk was higher in persons with DID alone (7.1%) and autism + DID (5.2%) when compared to a general population prevalence of 0.97% (Vivanti et al. 2021). There is sparse understanding of how persons with autism cope when parents are no longer available to support them into midlife or old age.

NDDs can be considered as chronic health conditions that require regular follow-up, routine screening, and interventions for medical and psychiatric issues. There has been encouraging evidence supporting better outcomes with specialist DID inpatient services compared to general adult psychiatric services (Xenitidis et al. 2004). “Dual diagnosis” services for persons with both DID and psychiatric disorders typically include functional communication training and positive behavioural support planning in HIC (Constantino et al. 2020). Outpatient, inpatient and community-based services that exclusively cater to the needs of persons NDD are almost non-existent in LAMIC.

## Recommendations

To enhance the care and services for adults with NDD, we propose the following recommendations that are applicable to worldwide, but specifically so for low-resource settings:

- Screening and diagnosis for multimorbidity should be routinely included in clinical care of persons with NDD in psychiatric settings. Structured assessment tools that are culturally validated should become a part of regular care when there are challenging behaviours, and these should be interpreted in the appropriate clinical context.
- Periodic audit of prescribing practices should be conducted to reduce polypharmacy and inappropriate use of medications. Locally relevant policies and guidelines need to be developed.
- Capacity building amongst physicians, psychiatrists and other multidisciplinary team members in general adult services should include strategies to enhance communication (verbal and non-verbal) while managing health issues in persons with NDD.
- Models of care for NDD that are relevant for LAMIC should be tested. These may necessitate integration of care and strong consultation-liaison across primary, secondary, and tertiary care. Tertiary care centres should develop specialized psychiatric services that cater to the complex mental health needs of adults with NDD.
- Virtual health care can provide care at the doorsteps for larger numbers and its potential needs to be explored. The COVID 19 pandemic has forewarned about the need to have such services in preparation for the future.
- Greater investment in research of multimorbidity in NDD across the lifespan should build the necessary evidence to improve services and quality of care for persons with NDD.
